# Labour and Neonatal Outcome in Small for Gestational Age Babies Delivered Beyond 36+0 Weeks: A Retrospective Cohort Study

**DOI:** 10.1155/2011/293516

**Published:** 2010-12-15

**Authors:** K. E. Boers, J. A. M. van der Post, Ben W. J. Mol, J. M. M. van Lith, S. A. Scherjon

**Affiliations:** ^1^Department of Gynaecology and Obstetrics, Bronovo Hospital, Bronovolaan 5, 2597 AX The Hague, The Netherlands; ^2^Department of Obstetrics, Academic Medical Centre, University of Amsterdam, 1105 AZ Amsterdam, The Netherlands; ^3^Department of Obstetrics, Leiden University Medical Centre, P.O. Box 9600, 2300 RC Leiden, The Netherlands

## Abstract

*Objective*. Small for gestational age (SGA) is associated with increased neonatal morbidity and mortality. At present, evidence on whether these pregnancies should be managed expectantly or by induction is lacking. To get insight in current policy we analysed data of the National Dutch Perinatal Registry (PRN). *Methods*. We used data of all nulliparae between 2000 and 2005 with a singleton in cephalic presentation beyond 36+0 weeks, with a birth weight below the 10th percentile. We analysed two groups of pregnancies: (I) with isolated SGA and (II) with both SGA and hypertensive disorders. Onset of labour was related to route of delivery and neonatal outcome. *Results*. Induction was associated with a higher risk of emergency caesarean section (CS), without improvement in neonatal outcome. For women with isolated SGA the relative risk of emergency CS after induction was 2.3 (95% Confidence Interval [CI] 2.1 to 2.5) and for women with both SGA and hypertensive disorders the relative risk was 2.7 (95% CI 2.3 to 3.1). *Conclusion*. Induction in pregnancies complicated by SGA at term is associated with a higher risk of instrumental deliveries without improvement of neonatal outcome. Prospective studies are needed to determine the best strategy in suspected IUGR at term.

## 1. Introduction

Intrauterine growth restriction (IUGR) and hypertensive disorders in pregnancy are important complications of pregnancy and are, also in term pregnancies, associated with an increased risk of maternal and perinatal morbidity and mortality [1–5]. At present there is evidence on the optimal treatment of pregnancies complicated by hypertension at term concerning the prevention of maternal morbidity [6]. However, evidence on the best management strategy for at term intrauterine growth restriction concerning neonatal outcome and labour process is still lacking. Dutch guidelines on the subject suggest either expectant management under strict monitoring of mother and child or induction of labour. On the one hand induction might preempt intrauterine fetal death. On the other hand induction of labour is thought to be associated with an increased rate of instrumental deliveries or emergency caesarean sections in retrospective studies [7–9]. Also neonatal outcome might be less favourable, related to induction of labour at a relatively early gestational age [10–12]. On the contrary, it has been demonstrated prospectively that induction of labour does not increase the risk for caesarean section while it reduces the risk of severe maternal morbidity [6]. Composite neonatal outcome in this study showed comparable neonatal outcome after induction and an expectant monitoring policy, but these children were not explicitly growth restricted [6]. To compare the neonatal outcome and intervention rates between induction and expectant monitoring of pregnancies complicated by growth restriction at term a multicentre randomised trial, the Disproportionate Intrauterine Growth Intervention Trial At Term (DIGITAT trial), was performed [13]. Prior to the results of this trial, we investigated current management policy on this subject in The Netherlands and analysed retrospectively data of the National Dutch Perinatal Registry (PRN) of pregnancies complicated by SGA at term to examine the onset of labour related to the mode of delivery and immediate neonatal outcome. 

## 2. Methods

In the National Dutch Perinatal Registry (PRN) a distinction is made between primary care by midwives of low-risk pregnancies (LVR1) and secondary and tertiary care by obstetricians for women with an increased perinatal risk (LVR2). We used data of the LVR2 between 2000 and 2005 to select those children delivered with a birth weight below the 10th percentile. In addition, we registered if these pregnancies were complicated by preeclampsia or gestational hypertension. Only nulliparae with a singleton pregnancy in cephalic presentation that ended after 36+0 weeks were included. We excluded women with pregnancies complicated by stillbirths as well as women who delivered a child with congenital abnormalities. Gestational hypertension was defined as diastolic blood pressure above 90 mmHg (Korotkoff V), measured at two occasions in normotensive women before pregnancy. Preeclampsia was defined as a diastolic blood pressure above 90 mmHg and proteinuria of at least 300 milligrams per 24 hours [14]. SGA was defined as a birth weight below the 10th percentile, according to the Dutch growth charts of Kloosterman [15]. Between January first 2000 and December 31st 2004 a total of 253.235 nulliparae with a singleton pregnancy delivered after 36+0 weeks under secondary and tertiary care. Of these 253.235 pregnancies 799 neonates died before delivery. Of the remaining 252.436 two groups of women were analysed: (i) 14.416 women who delivered a child with a birth weight below the 10th percentile without hypertension and (ii) 4574 women with pregnancies complicated by both IUGR and hypertensive disorders. Of all these women the onset of labour was recorded this was either a spontaneous onset, induction of labour with prostaglandins or amniotomy, or an elective caesarean section. In both groups of women onset of labour was related to the labour process (spontaneously, instrumental vaginal delivery and emergency or elective caesarean section) and to immediate neonatal outcome (intrapartum death, live birth with Apgar score <7 versus Apgar score ≥7 after 5 minutes). Adverse neonatal outcome was defined by neonatal outcome of 5-minute Apgar score <7 or intrapartum death. Both labour process and adverse neonatal outcome were primary outcomes of this retrospective study. Differences in the groups between the labour process and outcome were expressed as relative risks with confidence intervals of 95%. Statistical analysis was performed using SPSS software (version 16.0, Chicago, IL).

## 3. Results

A total of 14.416 normotensive women delivered a child with a birth weight below the 10th percentile (group I).  Table 1 shows the data of 14.294 women of whom both onset of labour and outcome of delivery, was known. Out of 14.347 pregnancies the immediate neonatal outcome as well as onset of labour was known (Table 2). 

Out of 4.574 women with pregnancies complicated by hypertensive disorders (preeclampsia or gestational hypertension) a child with a birth weight below the 10th percentile was born (group II).  Table 3 shows the results of the 4540 women of whom the onset of labour as well as route of delivery was known.  Table 4 displays the results of 4557 women of whom both onset of labour and immediate neonatal outcome were known. 

In both SGA groups, we found a higher risk of instrumental delivery after induction of labour with prostaglandins (Tables 1 and 3). We also found a higher risk of emergency caesarean section after induction of labour with oxytocine or amniotomy, but this was most obvious after priming with prostaglandins; in group I with isolated SGA the relative risk for emergency caesarean section was 2.3 (95% confidence interval (CI) 2.1 to 2.5) and in group II (IUGR complicated by preeclampsia or gestational hypertension) the relative risk for emergency caesarean was 2.7 (95% CI 2.3 to 3.1). Induction of labour with prostaglandines was not associated with an increased risk of adverse neonatal outcome. For the women with a combination of SGA and hypertensive disorders we found a higher risk of adverse neonatal outcome after elective caesarean section (RR 1.9; 95% CI 1.1 to 3.2).

## 4. Discussion

We examined a cohort of 18.990 women who delivered a child that was small for gestational age in the presence or absence of hypertensive disorders at term. In this cohort we found a distinct association between induction of labour and a higher risk of emergency caesarean sections. This association was most obvious in priming with prostaglandins. We also found a higher risk of instrumental deliveries after induction of labour, whereas induction did not improve the composed adverse neonatal outcome (5-minute Apgar score <7 and intrapartum death).

The strength of the present study is that analysis was performed on a large cohort of women who delivered a child with a birth weight below the 10th percentile. 

We did not find a benefit of inducing labour for isolated SGA nor for SGA with pregnancy-related hypertensive disorders for the immediate neonatal outcome. In pregnancies with a suspected growth restricted child, there are still doubts concerning the best policy [16]. Inducing labour might prevent possible perinatal morbidity and mortality, by freeing the growth restricted child from the undernourished environment. On the contrary observational studies showed that antenatal detection of growth restriction may be associated with an increased incidence of obstetric interventions, with no demonstrable positive effect upon the short-term neonatal outcome [17]. Also higher rates of preterm delivery are found mainly as a consequence of medical interventions to avoid fetal compromise in children with an antenatal diagnosis of intrauterine growth retardation [18]. 

Like other retrospective studies, we found that induction of labour in pregnancies, complicated by both SGA and hypertensive disorders in pregnancy, is associated with an increase in the caesarean section rate. In a study on induction of labour in primigravid women a doubling in the numbers of caesarean sections was found related to induction. This outcome was independent of the reason of induction [7]. This finding has to be weighted against the risk for complications for both mother and child in a next pregnancy [19]. However, most studies contain retrospective data, and there is evidence to doubt that these findings also apply in prospective trials [6]. Unfortunately, we can not exclude a possible selection bias, where the most severe cases (i.e., with the worse antenatal assessments) being induced with a less favourable cervix, as information on maternal and fetal condition as well as cervical condition are not registered in the PRN database. Moreover, one can only speculate about outcomes if these pregnancies would not have been induced but spontaneous onset of labour was awaited.

In our cohort we selected children retrospectively after they were born with a birth weight below the 10th percentile, so actually we selected children that were born small for gestational age. We cannot therefore automatically translate the results of this study to pregnancies in which IUGR is suspected antenatally by ultrasound. We were also not informed on ethnicity, which could be an important explaining variable for the different outcomes. It is well known that the use of customised growth curves results in a better selection of children who are actually growth restricted and in a better risk selection of perinatal mortality and morbidity [20]. In The Netherlands these curves are not generally applied and the PRN does not contain all items for the calculation of these customised curves retrospectively. 

In both groups of women direct neonatal outcome after elective caesarean section was remarkably less optimal than after a delivery that started vaginally. Fetal compromise after elective caesarean before 39 weeks of gestation might have been a contributing factor for that finding [21]. Furthermore, the occurrence of maternal hypotension due to spinal anaesthesia, leading to hypoperfusion of the placenta in an already compromised fetal condition, could be an explaining factor [22]. Moreover, we cannot exclude that elective caesarean section was performed in the most compromised pregnancies (e.g., with nonreassuring fetal heart rate) and subsequently represent a worse adverse outcome.

In a pilot study on pregnancies with IUGR at term it was found that it is feasible to randomise for this complication between immediate induction of labour or to a careful waiting policy until spontaneous delivery [23]. The study showed a randomisation to delivery interval of two weeks and an increase in mean birth weight of 100 grams in the expectant management group. No differences in obstetrical interventions and neonatal morbidity were found. This study was underpowered for neonatal outcome, and evidence on the best management strategy awaits prospective evaluation. The *DIGITAT trial (*Disproportionate Intrauterine Growth Intervention Trial At Term, ISRCT10363217) is investigating early induction versus expectant management in pregnancies complicated by IUGR at term, and results are underway [13]. The trial randomised 650 women and studied similar policies as the *HYPITAT trial* did for hypertension during pregnancy [6]. These trials are embedded in the Dutch Obstetric Consortium. Over 50 hospitals, academic and nonacademic, participated in these two trials (http://www.studies-obsgyn.nl). 

In conclusion, data collected via the National Dutch Perinatal Registry show that induction of labour is associated with an increased risk of emergency caesarean section and instrumental deliveries in pregnancies that delivered a child with a birth weight below the 10th percentile with or without preeclampsia or gestational hypertension at term. The risk on instrumental delivery is particularly high when labour is induced with prostaglandins. Compared to spontaneous delivery, induction of labour does not seem to improve the neonatal outcomes immediately after birth. However, these retrospective data represent outcomes in children selected after they are born with a low birth weight and should not be extrapolated to settings where growth restriction is suspected antenatally. 

Results of the DIGITAT trial, concerning not only medical outcomes but also cost, quality of life, and treatment preference analyses, as well as data of long-term neonatal followup will help to elucidate aspects of the best management strategy in IUGR at term.

## Figures and Tables

**Table 1:** Process of labour in pregnancies complicated by SGA.

	Route of delivery				RR (95% CI)	
Onset of labour	Spontaneous vaginal	Instrumental delivery	Emergency caesarean	Elective caesarean	Emergency caesarean	Instrumental delivery
Amniotomy 231 (2)	151	43	37	0	1.3 (0.95–1.7)	0.87 (0.73–1.0)
Oxytocine 1235 (9)	778	221	236	0	1.5 (1.3–1.7)	0.93 (0.86–1.0)
Prostaglandins 2191 (15)	1176	394	621	0	2.3 (2.1–2.5)	1.16 (1.1–1.2)
Spontaneous onset 10182 (71)	6125	2780	1277	0	ref	ref
Planned caesarean 455 (3)	0	0	0	455	n.a.	n.a.
Total 14.294/14.416	8230	3438	2171	455		

Displayed *n* (%). RR: relative risk; 95% CI: 95% confidence interval; ref: reference; n.a.: not appropriate

**Table 2:** Neonatal condition after birth in pregnancies complicated by SGA.

	Neonatal outcome			RR (95% CI)
	Intrapartum death	AS < 7	AS ≥ 7	AS < 7 or Intrapartum death
Amniotomy	0	7 (3.0)	224 (97.0)	0.96 (0.46–2.0)
Oxytocine	3 (0.2)	31 (2.5)	1202 (97.3)	0.88 (0.62–1.2)
Prostaglandins	5 (0.2)	53 (2.4)	2131 (97.4)	0.84 (0.64–1.1)
Spontaneous onset	30 (0.2)	291 (2.8)	9891 (97.0)	Ref
Planned caesarean	1 (0.2)	21 (4.4)	457 (95.4)	1.5 (0.96–2.2)
Total *n* = 14.347/14.416	39	403	13.905	

Displayed *n* (%). AS: Apgar-score after 5 minutes, RR: relative risk; 95% CI: 95% confidence interval; ref: reference; n.a.: not appropriate

**Table 3:** Process of labour in pregnancies complicated by SGA and hypertensive disorders (with or without proteinuria).

	Route of delivery				RR (95% CI)	
Onset of labour	Spontaneous vaginal	Instrumental delivery	Emergency caesarean	Elective caesarean	Emergency caesarean	Instrumental delivery
Amniotomy 112 (3)	63	26	23	0	1.6 (1.1–2.3)	1.1 (0.91–1.4)
Oxytocine 720 (16)	420	148	152	0	1.6 (1.3 – 1.9)	1.1 (0.97–1.2)
Prostaglandins 1733 (39)	872	280	621	0	2.7 (2.3–3.1)	1.3 (1.2–1.4)
Spontaneous onset 1558 (34)	959	394	205	0	ref	ref
Planned caesarean 377 (8)	0	0	0	377	n.a.	n.a.
Total 4540/4574	2314	848	1001	377		

Displayed *n* (%). RR: relative risk; 95% CI: 95% confidence interval; ref: reference; n.a.: not appropriate

**Table 4:** Neonatal condition after birth in pregnancies complicated by SGA and hypertensive disorders (with or without proteinuria).

	Neonatal outcome			RR (95% CI)
	Intrapartum death	AS < 7	AS ≥ 7	AS < 7 or Intrapartum death
Amniotomy	0	4 (4.0)	108 (96.0)	1.4 (0.50–3.7)
Oxytocine	0	23 (3.0)	697 (97.0)	1.2 (0.74–2.0)
Prostaglandins	2 (0.1)	42 (2.4)	1726 (97.5)	0.95 (0.62–1.4)
Spontaneous onset	1 (0.1)	40 (2.5)	1519 (97.4)	Ref
Planned caesarean	1 (0.2)	19 (4.8)	374 (95.0)	1.9 (1.1–3.2)
Total 4557/4574	4	128	4424	

Displayed *n* (%). AS: Apgar score after 5 minutes, RR: relative risk; 95% CI: 95% confidence interval; ref: reference; n.a.: not appropriate
